# Subscapular skinfold thickness, not other anthropometric and dual-energy X-ray absorptiometry-measured adiposity, is positively associated with the presence of age-related macular degeneration: a cross-sectional study from National Health and Nutrition Examination Survey 2005–2006

**DOI:** 10.1136/bmjophth-2023-001505

**Published:** 2024-07-31

**Authors:** Miguel Gedtal, Jayne Woodside, David Wright, Margaret Rayman, Ruth Esther Hogg

**Affiliations:** 1 Queen's University Belfast, Belfast, UK; 2 University of Surrey, Surrey, UK

**Keywords:** Epidemiology, Retina, Macula

## Abstract

**Objective:**

Current literature reveals an association between anthropometric measures of adiposity (AnthM) and age-related macular degeneration (AMD), but few have explored the disease association with imaging methods. This study aimed to explore the relationship between AMD status and dual-energy X-ray absorptiometry measures (DEXAMs) among a representative sample of the US population, and compare the association with AnthM.

**Method:**

Using a representative sample in the National Health and Nutrition Examination Study 2005–2006 (n=1632), DEXAMs across the whole body and waist (ie, android), and relative fat distributions (eg, percentage fat, android-to-total body ratio) were analysed between no AMD (baseline) and any AMD. Bivariate analyses across AMD status were similarly performed for AnthM (ie, body mass index, waist circumference and skinfold thicknesses) and potential confounders (ie, demographics and health-related variables). Significant adiposity measures were analysed using logistic regression, adjusting for confounders.

**Results:**

The participants in the sample were aged 40–69 years, were majority female (52%) and mainly Caucasian (76.5%). Bivariate analysis revealed having any AMD had positive significant associations with android-to-total fat ratio and subscapular skinfold thickness (SSFT). Other AnthM and DEXAMs were not significant. After adjusting age, gender and prescription of cholesterol-lowering medicine, only SSFT remained significantly associated.

**Conclusion:**

SSFT represents an independent risk factor for AMD presence compared with other AnthM and DEXAMs. SSFT is an established method of measuring fat under the skin (ie, subcutaneous fat). Hence, subcutaneous fat may be more relevant in explaining the adiposity–AMD link due to physiological properties specific to the tissue. Limitations include the restricted age range and low numbers of participants with late AMD.

**WHAT IS ALREADY KNOWN ON THIS TOPIC**

- The findings for the association between age-related macular degeneration (AMD) and obesity as measured by anthropometric measures are mixed. There are few epidemiological studies investigating the association between adiposity measured by dual-energy X-ray absorptiometry measures (DEXAMs) and AMD.

**WHAT THIS STUDY ADDS**

- In a representative US cross-sectional sample, AMD status was positively and significantly associated with subscapular skinfold thickness, which measures fat under the skin of the shoulder, but not other anthropometric and DEXAMs.

**HOW THIS STUDY MIGHT AFFECT RESEARCH, PRACTICE OR POLICY**

- The link between subcutaneous versus visceral fat would need to be explored and confirmed in a longitudinal study with a large sample of early/intermediate AMD and late AMD. Such investigations could clarify the adiposity–AMD link and provide guidance on specific lifestyle changes, for example, physical activity, which targets specific adipose depots, therefore effectively reducing the risk of incidence or AMD progression.

## Introduction

 Age-related macular degeneration (AMD) is a major cause of vision loss with an estimated 196 million people affected globally in 2020.^1^ Early or intermediate stages of the disease are usually asymptomatic, so the disease is often detected at a late stage.^2^ AMD risk factors can be categorised as non-modifiable, for example, genetics, or modifiable, for example, smoking, diet, adiposity and physical activity.^3^,^7^ Vision loss which accompanies late-stage AMD is generally irreversible; hence, a clear understanding of modifiable risks to enable primary or secondary prevention via behaviour modification would be a worthwhile goal. While obesity has been shown to induce AMD in animal models,^8^ evidence from human studies has been less straightforward. There are several means of identifying excessive adiposity. Anthropometric measures (AnthM) of anatomical proportions highlight excessive adiposity across the entire body [eg, body mass index (BMI)], in particular regions [eg, waist circumference (WC), waist/height ratio (WHR)] or measure adiposity deposited just under the skin (ie, skinfold thicknesses). Skinfold thicknesses across four regions (biceps, triceps, subscapular and iliac) are used to calculate body density and total fat.^9^ AnthM have been used to capture adiposity in large population-based studies.^10^ However, a recent systematic review by Ng Yin Ling *et al* found conflicting results in 16 epidemiological studies whereby AnthM (namely, BMI, WC and WHR) and incident AMD were positively associated in several prospective cohort studies, while others presented no or even inverse associations.^11^ Furthermore, AnthM including BMI have been criticised for their inability to distinguish fat versus lean mass in total mass hence leading to inaccurate measures of adiposity in individuals with high lean mass (eg, athletes), low lean mass (eg, elderly and those affected by sarcopenia, ie, loss of lean muscle mass) and for those with comorbidities such as oedema.^12 13^

Multislice MRI and CT are considered the reference standard for measuring total and regional adiposity in living participants^14 15^; however, they are costly and technically laborious with CT requiring radiation exposure thus preventing their use in large-scale studies.^16^ Another imaging technique uses dual-energy X-ray absorptiometry measure (DEXAM) which uses much lower (and thus safer) levels of radiation, is low-cost and easier to operate. Several cross-sectional studies have found strong agreement between DEXAM and MRI; DEXAM is therefore a valid alternative to measure adiposity.^17^ Furthermore, DEXAM differentiates between tissue (lean vs fat) types and captures the mass of each tissue type, unlike AnthM which are proxy measures of adiposity and may be inaccurate on some occassions.^13^ Excessive dual-energy X-ray absorptiometry (DEXA)-measured android adiposity (that is, fat around the waist) is deemed particularly harmful as it has been positively associated with diabetes and cardiovascular disease: greater android adiposity may impair insulin resistance, glucose tolerance and unfavourably affect levels of high-density lipoproteins (HDLs), low-density lipoproteins (LDLs) and triglycerides.^18^ An aetiological effect of android fat on AMD may exist: one case–control study reported that patients with late AMD compared with matched controls had significantly higher BMI and DEXAM of central-abdominal-to-total body fat ratio.^19^ However, the number of cases was small (n=54) and all had late-stage AMD; thus, the association may be different in a larger sample including those with asymptomatic, early/intermediate-stage AMD.

To our knowledge, there are limited studies that have compared DEXAM, particularly android fat, among those with and without AMD in a population setting or explored whether there are differences in the association between AnthM and DEXAM with AMD. The National Health and Nutrition Examination Survey (NHANES) collected and graded retinal images for the presence of AMD in a representative sample of older adults in the USA (2005–2006) and undertook DEXAM in a subsample thus enabling exploration of the association between DEXAM and AMD.

## Materials and method

### Study design

NHANES is an ongoing survey conducted by the National Center for Health Statistics which evaluates a representative sample of non-institutionalised US civilians. Participants are selected by a complex, multistage probability design.^20^ Survey personnel ﬁrst interview participants in their homes during which interviewers collect information on demographic characteristics and health-related issues. One to 2 weeks after the interview, participants undergo a standardised physical examination and blood collection in a mobile examination centre, and dietary recall information is collected. A second dietary recall is conducted via telephone 3–10 days after the ﬁrst recall. Both recalls are collected by using the US Department of Agriculture’s Automated Multiple Pass Method.^21^ Data from the 2005–2006 NHANES (https://wwwn.cdc.gov/nchs/nhanes/search/datapage.aspx?Component=Examination&CycleBeginYear=2005)^22^ were used as this is the only NHANES cycle with both colour fundus photographs, retinal feature grading and DEXAM.^23 24^

### Assessment of AMD

Detailed procedures of the retinal examination performed in the NHANES 2005–2006 can be found elsewhere (https://wwwn.cdc.gov/Nchs/Nhanes/2005-2006/OPXRET_D.htm). Fundus photographs were captured using Canon Non-Mydriatic Retinal Camera CR6-45NM. No pharmacological dilation was used but the room was darkened to encourage maximal pupil dilation. The first image was centred on the macula (field 2) and the second on the optic nerve (field 1). Retinal photographic grading of both eyes of each participant was conducted by two senior graders using a modified Wisconsin Age-Related Maculopathy Grading System protocol. Further details can be found elsewhere (http://www.cdc.gov/nchs/data/nhanes/nhanes_05_06/NHANES_ophthamology_digital_grading_protocol.pdf). To enable comparisons with other recent analyses, the grading data were translated into an AMD severity stage using an adaptation of the Beckman Institute’s classification system to identify participants with early, intermediate and late AMD in the worse eye as has been applied elsewhere.^2 25^ Controls were identified if the worse eye had neither drusen nor pigmentary abnormalities or the worse eye had pigmentary changes (retinal pigment epithelium (RPE) hyper/hypopigmentation) either alongside hard drusen or no drusen. Early AMD was defined by the absence of geographical atrophy and exudative AMD and the presence of soft drusen with no pigmentary changes. Intermediate AMD was defined by the absence of geographical atrophy and exudative AMD, and the presence of large drusen (≥125 µm^25^) with or without pigmentary abnormalities. Late AMD was defined by the presence of exudative AMD or geographical atrophy.^2^ Participants were excluded if they were under 40 years of age, had eye infections, had no light perception in either eye or had eye patches on both eyes.

### Dual-energy X-ray absorptiometry measures

Whole-body DEXAMs in NHANES 2005–2006 were performed using Hologic QDR-4500A fan-beam densitometer (Hologic, Bedford, Massachusetts, USA). Exclusion criteria for DEXAM were ages <8 or >69 years, pregnancy, self-reported history of radiographic contrast material use in the past 7 days, self-reported nuclear medicine studies in the past 3 years and individuals with self-reported weight over 300 lbs (136.1 kg) or a standing height over 6’5” (195.6 cm) as they did not fit into the DEXA machine. DEXAMs of whole body bone mineral content, bone mineral density, per cent fat, lean mass, fat mass and regional measurements (eg, arm, leg and trunk) were collected. Owing to non-random missing values, NHANES researchers undertook several imputations (n=5) for the regional and whole-body measures (except for android or gynoid fat). The appendicular skeletal muscle mass was derived from the sum of lean mass in both legs and arms. Then, sarcopenia was determined by cut-off points <20 kg for men and <15 kg for women.^26^ Android and gynoid regions were defined by the Hologic APEX software used in the scan analysis. The android region is the area around the waist between the midpoint of the lumbar spine and the top of the pelvis. The gynoid is situated between the head of the femur and the mid-thigh. DEXAMs used in this study were the following: total body fat mass, percentage of fat in total mass, android fat mass, android-to-total fat mass and android fat percentage. DEXA-measured lean mass in the limbs was also acquired to determine sarcopenia diagnosis: sarcopenia is known to affect AnthM.^13^

### Covariates selection and preparation

**Figure 1:**
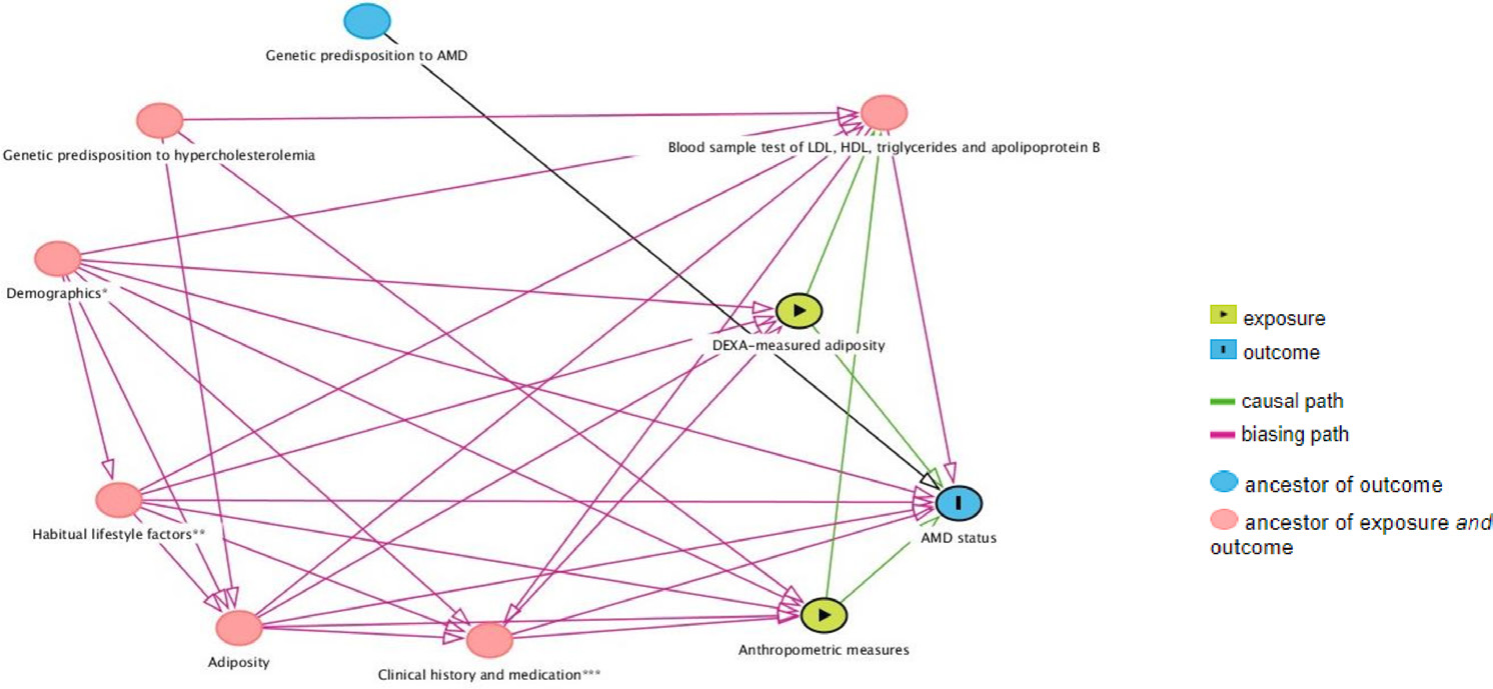
Directed acyclic graph for covariate selection. *Demographics indicate age; sex; self-reported variables annual household income (under vs above $45 000); poverty/income ratio; ethnicity (Caucasian vs not Caucasian); educational attainment (up to high school vs any higher qualification). **Habitual lifestyle factors indicate physical activity (vigorous activity and leisure activities performed in the previous month); the number of cigarettes smoked (self-reported cigarettes smoked in lifetime equals 100 or not); the number of days where at least one alcohol drink was taken in the past year, with one drink equating to a 12 oz beer, a 5 oz glass of wine or 1.5 oz of liquor; usual intake for monounsaturated, saturated, polyunsaturated fats and zinc. ***Clinical history and medication indicate the clinical history of high cholesterol/hypercholesterolaemia (never clinically tested cholesterol levels vs history of clinically diagnosed high cholesterol vs no history of high cholesterol); self-reported history of receiving a prescription of cholesterol-lowering medication; hyperglycaemia (glycohaemoglobin percentage at the cut-off point ≥6.5% or fasting glucose of ≥126 mg/dL). Note that the HDL, LDL and triglycerides in the blood sample occur after the exposure and are mediators to the outcome. However, serum triglyceride and lipoprotein levels were selected as a means of controlling the genetic effects that determine hypercholesterolaemia; such effects could confound the exposure–outcome relationship. Ultimately, gender, age and potential confounders statistically proven to be associated with the outcome (ie, AMD status) upon bivariate analysis were controlled during multivariate analysis. AMD, age-related macular degeneration; DEXA, dual-energy X-ray absorptiometry; HDL, high-density lipoprotein; LDL, low-density lipoprotein.

The following variables that measure adiposity of the total body or at the trunk region were available and selected for the study: BMI in kg/m^2^, WC in cm and available skinfold thicknesses (subscapular and triceps) in mm. Skinfold thicknesses for bicep and superiliac were unavailable; hence, total body fat using the skinfold thicknesses could not be calculated using the available dataset. A directed acyclic graph was used to depict the potential causal relationship between AnthM or DEXAM (exposure) and AMD status (outcome), and the observed and unobserved covariates that affect both exposure and outcome as a means to justify covariate selection as has been done previously^27^ (figure 1). The covariates selected are thought to be confounders which could bias the exposure–outcome relationship based on previous literature.^3^,^6^ Since adiposity and AMD status were captured at a one-time point during the physical examination in the mobile examination centre, characteristics or behaviours in the month or year before the participant’s examination were primarily selected to avoid controlling for mediators (ie, covariates that proceed after an exposure/outcome) which could induce collider bias.^28^ Ultimately, selected covariates could be categorised as follows: demographics [age, sex, self-reported variables annual household income (under vs above $45 000), poverty/income ratio, ethnicity (Caucasian vs not Caucasian), educational attainment (up to high school vs any higher qualification)]; clinical variables (clinical history of high cholesterol/hypercholesterolaemia (never clinically tested cholesterol levels vs history of clinically diagnosed high cholesterol vs no history of high cholesterol), self-reported history of receiving a prescription of cholesterol-lowering medication, non-fasting levels of HDL and glycohaemoglobin percentage, and fasting levels of LDL, triglycerides and apolipoprotein B); and habitual lifestyle factors [physical activity (vigorous activity and leisure activities performed in the previous month), the number of cigarettes smoked (self-reported cigarettes smoked in lifetime equals 100 or not), the number of days were at least one alcohol drink was taken in the past year (one drink equating to a 12 oz beer, a 5 oz glass of wine or 1.5 oz of liquor), and usual intake of nutrients (monounsaturated, saturated, polyunsaturated fats and zinc)]. Glycohaemoglobin percentage at the cut-off point ≥6.5% or fasting glucose of ≥126 mg/dL was considered as hyperglycaemia which is an indicator of diabetes.^29^ Usual intake for monounsaturated, saturated, polyunsaturated fats and zinc was calculated from 2-day dietary recalls between those by acquiring the best linear unbiased predictor of nutrient intake in those with no AMD and with any AMD using PC-Side (V.1.0).^30^

### Data analysis

Statistical analyses were performed using procedures from sample survey data using R V.4.3.0^31^ to account for the complex survey design used in NHANES. The survey package V.4.1-1 was used to generate weighted estimates.^32^ Weights accounting for measurements in the mobile examination centre, 2-day dietary recall and blood tests were used to provide nationally representative estimates.

To determine the robustness of significant findings and to avoid bias from missing data, missing data for relevant AnthM and other covariates were imputed (iterations=30). A master dataset of NHANES 2005–2006 of >400 variables (ie, extensive demographic information, physical examination results, laboratory results and questionnaire items) was used to select predictors for imputation.^33^ Self-reported history of receiving a prescription of cholesterol-lowering medication, physical activity (vigorous activity and leisure activities performed in the previous month), the number of cigarettes smoked (self-reported cigarettes smoked in lifetime equals 100 or not), the number of days in which at least one alcohol drink was taken in the past year and android fat mass were added in the master dataset. The method of predictor selection for imputation has been detailed elsewhere.^34^ In brief, covariates with high proportions of missing data (>50%), administrative variables, constant variables and variables that correlate higher than 0.999 with another variable were removed.^34^ Proportion, mean, SD and quartiles (median, first and third quartile) for no AMD and any AMD were measured for each imputation accounting for the complex sampling design of the NHANES. Apart from age and gender, variables not statistically associated with AMD status did not proceed to multivariate analysis. Furthermore, logistic regressions were performed with AMD status as the dependent variable. Adiposity measures were independently modelled as independent variables alongside confounders for each imputation. Estimates were subsequently pooled using the mitools package V.2.4.^35^ Associations between risk of AMD presence and each variable were expressed as ORs. P values following multivariate analysis and bivariate analysis, namely, t-tests (for means), Wilcoxon rank tests (for medians) and Χ^2^ tests (for proportion) between AMD status were acquired for each imputation. Pooled p values were derived using the median-p-rule, that is, the median p value across the iterations.^36^ P values were considered significant when p<0.05. For sensitivity analysis, we analysed complete case analysis without multiple imputation.

## Results

Of the 10 348 individuals in the NHANES 2005–2006 survey, 2934 had retinal imaging available to be graded using the adapted Beckman staging system. Of those with Beckman AMD gradings, 1632 had DEXAM and had an android adiposity scan available. The final sample ranged in age between 40 and 69 years [median age (IQR)=51 (13)], was predominantly female (52%) and Caucasian (76.5%). There were 1451 (raw percentage=88.9%) with no AMD and 181 (11.1%) had any AMD. Among those with any AMD, only two had late AMD (0.01%). Those with no AMD when compared with participants with any AMD were younger (51 years vs 54 years,) reported fewer individuals prescribed cholesterol-lowering medication (21% vs 32%) and reported lower adiposity (online supplemental table). Specifically, those with no AMD had significantly lower measures for subscapular skinfold thickness (SSFT) (23 mm vs 24 mm) and android fat-to-total fat ratio (0.081 vs 0.085) relative to those with any AMD (online supplemental table). Similar associations were revealed upon sensitivity analysis except for android-to-total fat ratio which was not found significant. WC was not significant but approaching significance in the expected direction for any AMD (p=0.051). The remaining AnthM [BMI and skinfold thicknesses (triceps and subscapular)], DEXAM (android fat mass and percentage; total fat mass and percentage; android-to-gynoid ratio), health-related variables and demographics did not significantly differ upon bivariate analysis. After adjusting for age and sex, the positive associations were lost between android-to-total fat and having any AMD; the positive association remained for SSFT (table 1). After controlling additionally for self-reported history of cholesterol-lowering medication, the positive association of SSFT with any AMD persisted so that an increase of SSFT by 1 mm increased risk of any AMD by 3%, holding age, gender and history of cholesterol prescription constant.

**Table 1:** Minimally and fully adjusted models to determine association of adiposity measures and AMD status

Variable	Minimally adjusted			Fully adjusted		
	ß	95% CI	P value	ß	95% CI	P value
No AMD vs any AMD						
Android-to-total fat ratio*	7.858	−3.597, 19.313	0.193	6.199	−6.101, 18.5	0.332
Subscapular skinfold thickness, mm	0.030	0.007, 0.053	0.017†	0.027	0.004, 0.051	0.033†

Covariates included in the minimally adjusted model: age+sex.

Covariates included in the fully adjusted model: age+sex+history of cholesterol-lowering prescription. Adiposity measures were modelled separately by logistic regression for each AMD status change.

* The ß and CIs of android-to-total fat ratio were measured after pooling across five imputations already available in the dataset. The pooled p value was acquired using the median-p value rule. Appropriate weights and information on the complex sampling design of the National Health and Nutrition Examination Survey were used to provide nationally representative estimates of the US population.

† Significant p values (p<0.05).

ßbeta-coefficientAMDage-related macular degeneration

## Discussion

In this representative sample of adults in the USA aged 40–69 years, slightly thicker SSFT was significantly associated with modest increases in risk of any AMD even when age, gender and history of cholesterol prescription were controlled. Specifically, as SSFT increases by 1 mm, the risk of AMD increases by 3%. The risk of selection bias was addressed by using sampling weights and imputing for missing values. To our knowledge, there have been no studies that have found a significant association between SSFT and AMD status. Studies have measured WC (which was borderline significant for this study) and WHR^11^; both WC and WHR capture variations in subcutaneous fat.^37^ Peeters *et al* reported that in a large sample of adults in the USA (baseline: 45–64 years), a decrease in WHR from baseline was significantly associated with lower odds of any AMD and any incident abnormalities in the RPE.^38^ SSFT is strongly associated with subcutaneous fat in the trunk,^39^ and similarly, there is some evidence that WHR reflects subcutaneous fat in the abdomen and trunk more sensitively than other AnthM such as WC.^37 40^ Hence, subcutaneous fat in the trunk may play a more significant role in the AMD–adiposity link.

This is highlighted by how android fat mass captured by DEXA was not associated with early/intermediate or any AMD in this study. Android fat reflects both subcutaneous fat and visceral fat which lies beneath the subcutaneous fat and around internal organs. Furthermore, derived measures of adiposity in the android which aim to capture visceral fat (ie, android–gynoid percentage ratio and android-to-total fat mass) were not associated with AMD. This contrasts with findings from Haas *et al*’s case–control study which found a significant association between AMD status and android–total fat ratio, though their sample was small (n=24) and all of their cases had exudative AMD.^19^ It is well recognised that risk factors for AMD differ between early/intermediate and late AMD stages.^41^ We had limited our sample in NHANES 2005–2006 to those <69 years as this was the age cut-off for DEXAM, resulting in a limited sample with late AMD (n=2). In another study of NHANES 2005–2006, the mean age of those with late AMD was 79.2 years (n=17).^42^ Thus, comparison with Haas *et al*’s sample with exudative AMD is limited; we had a smaller number of late AMD and primarily had patients with early/intermediate AMD. No measures of subcutaneous fat were performed by Haas *et al*, further limiting comparisons with this current study. The link between subcutaneous versus visceral fat would need to be explored and confirmed in a longitudinal study with a large sample of early/intermediate AMD and late AMD.

Several hypothesised mechanisms may explain the association between AMD and adiposity: excessive fat may induce abnormal inflammation in the retina or sequester antioxidants away from the eye.^7 8 43^ The ‘oil spill’ theory states that an abnormal influx of lipoproteins in the eye may produce drusen which are characteristic of AMD; therefore, excessive adiposity contributes to elevated AMD risk through this mechanism.^44^ Expression of certain genes could explain the AMD–adiposity link. Among obese mice, it was found that aberrant genetic expression by macrophages resulted in retinal damage directly or through systemic inflammation.^8 45^ Nonetheless, the main focus of animal studies has been on visceral fat but not subcutaneous fat. Subcutaneous fat and visceral fat are physiologically different: several groups have reported differential gene expressions of abdominal subcutaneous and visceral fat cells among humans.^46 47^ SERPINA5, a proinflammatory and coagulation-modulating gene, is expressed 17-fold greater in the subcutaneous abdominal tissue relative to visceral abdominal tissue^47^; significant upregulation of SERPINA5 has been also found in eyes at early stages of AMD.^48^ Subcutaneous fat seems to be more associated with HDL expression and modulation compared with visceral fat^47 49^; again, this may play a part in AMD onset and development. Furthermore, symptoms of a rare disease called partial lipodystrophy involve the abnormal distribution of subcutaneous fat in the face, trunk and limbs. Case reports of partial lipodystrophy with excessive adiposity in the trunk have been accompanied by widespread drusen and both dry and late AMD.^50 51^

Limitations of this study include the restricted age range which would have restricted the number of participants with late AMD. Furthermore, the mean BMI of the samples for no AMD and any AMD samples reached the cut-off for overweight; hence, the findings may only apply to overweight participants. Nonetheless, the sample was a representative US sample of older adults. Other strengths include the imputation of missing covariates during the study to lessen bias from missing values; standardised objective methods for assessing AMD and DEXAM; quality control of body measurements including training and calibration for the use of skinfold callipers [BM.pdf (cdc.gov)BM.pdf (cdc.gov)]; and the assessment of comprehensive demographic characteristics and health indicators. Nonetheless, direct DEXA-measured subcutaneous fat and the WHR were unavailable or could not be calculated in the NHANES 2005–2006 cycle. Regardless of our attempts to reduce collider bias and reverse causation, some of the biases are inherently extant in a cross-sectional study. To establish causality, the incidence or progression of AMD and changes in subcutaneous and/or visceral fat in the trunk across a large prospective cohort should be investigated.

In conclusion, our study suggests that SSFT, an established method of measuring subcutaneous adiposity, is associated with the presence of AMD compared with other DEXAMs or other AnthM. Hence, subcutaneous fat may be more relevant in the adiposity–AMD link. Further studies should investigate the relationship, if any, between the incidence or progression of AMD and changes in subcutaneous and/or visceral fat in the trunk across a large prospective cohort with early/intermediate and late AMD. This investigation could clarify the adiposity–AMD link and provide guidance on specific lifestyle changes, for example, physical activity, which targets specific adipose depots, therefore effectively reducing the risk of incidence or AMD progression.

## supplementary material

10.1136/bmjophth-2023-001505 online supplemental table 1

10.1136/bmjophth-2023-001505 online supplemental table 2

10.1136/bmjophth-2023-001505 online supplemental file 1

## Data Availability

Data are available in a public, open access repository.
