# Supplementary material for: Subscapular skinfold thickness, not other anthropometric and dual-energy X-ray absorptiometry-measured adiposity, is positively associated with the presence of age-related macular degeneration: a cross-sectional study from National Health and Nutrition Examination Survey 2005–2006
Source: BMJ Open Ophthalmol. 2024 Jul 31;9(1):e001505. doi: 10.1136/bmjophth-2023-001505 (PMC11293401; doi:10.1136/bmjophth-2023-001505)
Supplement: online supplemental table 1 [file bmjophth-9-1-s001.pdf]

Variable	No AMD in worse eye([a]=1451, [b]=690, [c]=1335)	Any AMD in worse eye([a]=181, [b]=90, [c]=163)	p-value
	mean (SD), median (IQR) or minimum-maximum count(%).Sample size for examination weights([a]), fasting weights([b]) and dietary recall weights([c]) differ.		
**Demographics([a])**			
Age, years	51 (IQR=13)	54 (IQR=16)	<0.001*
Gender			
Male	716 (47.2%)	102 (54.8%)	0.126
Female	735 (52.8%)	79 (45.2%)	0.126
Ethnicity			
Not Caucasian	716 (23.3%)	98 (25.7%)	0.534
Caucasian	735 (76.7%)	83 (74.3%)	0.534
Poverty Income Ratio	3.79 (IQR=3)	3.169 (IQR=3)	0.255
Annual family income			
Family income under $45k	747-751 (36.7%)	73-75 (47.5%)	0.096
Highest education attained			
Up to high school	695-696 (39.4%)	89 (40.3%)	0.865
**Measurements from DEXA([a])**			
Android-to-total fat ratio [d]	0.081 (+/-0.001)	0.085 (+/-0.003)	0.039*
Total fat % [d]	34.744 (+/-0.566)	35.268 (+/-1.037)	0.269
Android fat mass, g	2470.817 (+/-115.175)	2661.178 (+/-240.948)	0.144
Android fat %	34.606 (+/-0.699)	35.771 (+/-1.215)	0.076
Android-to-gynoid ratio	1.064 (+/-0.013)	1.099 (+/-0.033)	0.068
Total fat mass, g [d]	27616.08 (IQR=13939.66)	28140.3 (IQR=15518.34)	0.107
Has sarcopenia [d]	210-212(14.7%)	25-26(12.9%)	0.675
**Anthropometric measurements([a])**			
Body Mass Index, kg/m^2	28.765 (+/-0.514)	29.843 (+/-1.229)	0.098
Waist circumference, cm	98.902 (+/-1.364)	102.178 (+/-3.063)	0.051
Subscapular skinfold thickness, mm	22.758 (+/-0.681)	24.334 (+/-1.399)	0.017*
Tricep skinfold thickness, mm	20.578 (+/-0.561)	21.457 (+/-1.24)	0.123
**Clinical variables([a,b])**			
HDL, mg/dL([a])	55.155 (+/-0.954)	55.083 (+/-2.735)	0.953
Triglycerides, mg/dL([b])	152.845 (+/-13.164)	140.11 (+/-28.818)	0.514
LDL, mg/dL([b])	122.574 (+/-2.416)	119.847 (+/-7.437)	0.532
Apolipoprotein B, mg/dL([b])	105.314 (+/-3.36)	104.792 (+/-6.114)	0.916
Self-reported history of hypercholesterolaemia([a])			
Never had cholesterol checked	270-276 (13.8%)	27-28 (10.2%)	0.318
History of high cholesterol	543-550 (38.5%)	70-73 (41.5%)	0.318
Cholesterol tested with no hypercholesterolaemia	629-634 (47.8%)	81-83 (48.3%)	0.318
Self-reported history of receiving cholesterol-lowering prescription([a])			
Has been prescribed cholesterol-lowering medicine	308-312 (20.7%)	52 (31.6%)	0.001*
Has hyperglycaemia([b])	102-103 (9.2%)	18-19 (17.2%)	0.076
**Habitual lifestyle factors([a,c])**			
Total MET score of activities in the past 30 days([a])	7 (IQR=10)	5.133 (IQR=10)	0.16
Has smoked 100 cigarettes in lifetime([a])	764 (51.8%)	105 (59.5%)	0.083
Has done vigorous exercise in the past 30 days([a])	438 (34.2%)	44 (28.6%)	0.204
Total days with at least one alcoholic drink taken in the past year([a])	7.033 (IQR=23)	7 (IQR=16)	0.352
Monounsaturated fat usual intake, g/day([c])	30.878 (+/-0.353)	30.882 (+/-1.313)	0.998
Saturated fat usual intake, g/day([c])	27.966 (+/-0.352)	28.567 (+/-1.366)	0.689
Polyunsaturated fat usual intake, g/day([c])	17.569 (+/-0.178)	17.277 (+/-0.607)	0.66
Zinc usual intake, mg/day([c])	12.541 (+/-0.146)	12.204 (+/-0.358)	0.469

AMD= Age-related-macular degeneration; IQR= Inter-quartile range; DEXA= Dual X-ray Absorptiometry; HDL= High-density lipoprotein; LDL= Low-density lipoprotein; MET score= Metabolic score. *= Significant p-values (<0.05). The total sample for each group varied depending on the availability of the following weights: examination weights([a]), dietary recall weights ([c]) and fasting weights([b]). [d] The estimates of a given covariate were acquired by pooling across the five imputations performed by researchers in the National Health and Nutrition Examination Survey (NHANES). The pooled p-value was acquired using the median-p-value rule. The minimum-maximum of the imputed count for a given proportion may be the same, as are for covariates with no missing observations, hence the count is presented once in such cases. Appropriate weights and information on the complex sampling design of the NHANES were used to provide nationally representative mean, median, proportion, percentages and p-values of the US population.
