# Supplementary material for: Subscapular skinfold thickness, not other anthropometric and dual-energy X-ray absorptiometry-measured adiposity, is positively associated with the presence of age-related macular degeneration: a cross-sectional study from National Health and Nutrition Examination Survey 2005–2006
Source: BMJ Open Ophthalmol. 2024 Jul 31;9(1):e001505. doi: 10.1136/bmjophth-2023-001505 (PMC11293401; doi:10.1136/bmjophth-2023-001505)
Supplement: online supplemental table 2 [file bmjophth-9-1-s002.pdf]

	No AMD		Any AMD		
**Variable**	**mean (SD), median (IQR) or proportion (%)**	**missing from analysis depending on wts used (EW=1,451; DRW= 1,335; FW= 690)**	**mean (SD), median (IQR) or proportion (%)**	**missing from analysis depending on wts used (EW=181; DRW= 163; FW= 90)**	**p-value**
**DEMOGRAPHICS**					
**Age, years**	51 (IQR=13)	0	54 (IQR=16)	0	<0.001*
**Gender**					
**Male**	716 (41.9%)	0	102 (64.4%)	0	0.126
**Female**	735 (58.1%)	0	79 (35.6%)	0	
**Ethnicity**					
**Not Caucasian**	716 (26.4%)	0	98 (20%)	0	0.534
**Caucasian**	735 (73.6%)	0	83 (80%)	0	
**Poverty Income Ratio**	3.79 (IQR=2.91)	46 (3.2%)	3.18 (IQR=3.01)	6 (3.3%)	0.289
**Annual family income**					
**Family income under $45k**	680 (38.5%)	36 (2.5%)	106 (45.8%)	2 (1.1%)	0.097
**Highest education attained**					
**Up to high school**	695 (37.3%)	1 (0.1%)	89 (21.1%)	0	0.858
**MEASUREMENTS FROM DEXA**					
**Android-to-total fat ratio**	0.082 (+/-0.001)	1162 (80.1%)	0.087 (+/-0.006)	150 (82.9%)	0.526
**Total fat %**	35.491 (+/-0.583)	1162 (80.1%)	34.172 (+/-2.649)	150 (82.9%)	0.463
**Android fat mass, g**	2702.182 (+/-109.939)	0	2964.702 (+/-418.503)	0	0.144
**Android fat %**	35.807 (+/-0.626)	0	35.451 (+/-2.401)	0	0.076
**Android-to-gynoid ratio**	1.062 (+/-0.015)	0	1.183 (+/-0.07)	0	0.068
**Total fat mass, g**	34202.6 (IQR=18829.7)	1162 (80.1%)	32983.8 (IQR=18008.4)	150 (82.9%)	0.904
**Has sarcopenia**	19 (7.2%)	1162 (80.1%)	3 (8.3%)	150 (82.9%)	0.813
**ANTHROPOMETRIC MEASURES**					
**Body Mass Index, kg/m2**	29.977 (+/-0.54)	4 (0.3%)	31.624 (+/-1.562)	0	0.096
**Waist circumference, cm**	102.233 (+/-1.328)	4 (0.3%)	107.894 (+/-3.426)	1 (0.6%)	0.054
**Subscapular skinfold thickness, mm**	24.197 (+/-0.645)	358 (24.7%)	23.738 (+/-1.539)	52 (28.7%)	0.024*
**HDL, mg/dL**	55.925 (+/-2.191)	39 (2.7%)	52.231 (+/-3.811)	5 (2.8%)	0.978
**Triglycerides, mg/dL**	132.21 (+/-3.004)	22 (3.2%)	125.104 (+/-7.389)	3 (3.3%)	0.505
**LDL, mg/dL**	121.446 (+/-1.445)	53 (7.7%)	118.129 (+/-3.102)	5 (5.6%)	0.339
**Apolipoprotein B, mg/dL**	103.933 (+/-1.705)	22 (3.2%)	103.331 (+/-2.67)	3 (3.3%)	0.886
**Self-reported history of hypercholesterolaemia**					
**Never had cholesterol checked**	261 (11.9%)	45 (3.1%)	27 (0.5%)	4 (2.2%)	0.38
**History of high cholesterol**	531 (43.1%)	45 (3.1%)	70 (38.7%)	4 (2.2%)	
**Cholesterol tested with no hypercholesterolaemia**	614 (45.1%)	45 (3.1%)	80 (60.8%)	4 (2.2%)	
**Self-reported history of receiving cholesterol-lowering medicine**					
**Has been prescribed cholesterol-lowering medicine**	306 (25.1%)	45 (3.1%)	52 (37.3%)	4 (2.2%)	0.001*
**Has hyperglycaemia**	101 (9.2%)	13 (1.9%)	16 (17.2%)	3 (3.3%)	0.076
**HABITUAL LIFESTYLE FACTORS**					
**Total MET score of activities in the past 30 days**	10 (IQR=14)	547 (37.7%)	10.5 (IQR=14.5)	79 (43.6%)	0.398
**Has smoked 100 cigarettes in lifetime**	764 (47.2%)	1 (0.1%)	105 (51.9%)	0	0.083
**Has done vigorous exercise in the past 30 days**	438 (32.4%)	0	44 (37.7%)	0	0.204
**Total days with at least one alcoholic drink taken in the past year**	10 (IQR=22)	174 (12%)	7 (IQR=20)	23 (12.7%)	0.288
**Monounsaturated fat usual intake, g/day**	30.878 (+/-0.353)	0	30.882 (+/-1.313)	0	0.998
**Saturated fat usual intake, g/day**	27.966 (+/-0.352)	0	28.567 (+/-1.366)	0	0.689
**Polyunsaturated fat usual intake, g/day**	17.569 (+/-0.178)	0	17.277 (+/-0.607)	0	0.66
**Zinc usual intake, mg/day**	12.541 (+/-0.146)	0	12.204 (+/-0.358)	0	0.469
